# Case report: Overlapping anti-AMPAR encephalitis with anti-IgLON5 disease post herpes simplex virus encephalitis

**DOI:** 10.3389/fimmu.2023.1329540

**Published:** 2024-01-08

**Authors:** Shihui Sun, Jiafeng Ren, Zhao Zhong, Xuxia Ma, Danqing Shang, Changjun Su, Xianchao Zhao

**Affiliations:** Department of Neurology, the Second Affiliated Hospital of Air Force Military Medical University, Xi’an, Shaanxi, China

**Keywords:** autoimmune encephalitis, herpes simplex virus 1, overlap, anti-AMPAR encephalitis, anti-IgLON5 disease

## Abstract

Autoimmune encephalitis (AE) is the result of an autoimmune process that occurs as a rapidly advancing encephalopathy. Autoimmune encephalitis was commonly linked to herpes simplex virus 1 (HSV-1) as the most frequently identified virus. The main areas affected by this invasion are the temporal lobe, frontal lobe, and limbic system. Limbic encephalitis is a highly uncommon occurrence involving anti-alpha-amino-3-hydroxy-5-methyl-4-isoxazolepropionic acid receptor (AMPAR) encephalitis and anti-IgLON family member 5 (IgLON5) disease, both belonging to the rare category. As far as we know, this is the first report showing that a patient diagnosed with AMPAR encephalitis overlapped with anti-IgLON5 disease post herpes simplex virus encephalitis (HSE), which helps to broaden the range of this uncommon autoimmune disease. We recommend autoantibody testing in all patients with HSE, particularly those involving neurological relapses or progression.

## Introduction

Autoimmune encephalitis (AE) is a series of immune-mediated neurological disorders. At present, AE accounts for 10% to 20% of encephalitis cases. Some AE cases have prodromal infection-like symptoms following infection due to the herpes simplex virus 1 (HSV-1) (1), Epstein–Barr virus (2), cytomegalovirus, varicella zoster virus (3), and Coronavirus disease 2019 (4). Patients with AE are positive for one or more antibodies against neuronal cell-surface proteins and synaptic receptors, mainly anti-NMDAR antibodies accounting for around 80%. Venkatesan et al. reported that AE was also linked to HSV-1 as the primary cause of encephalitis (5). The clinical course of herpes simplex virus encephalitis can be concurrent with AE and is usually characterized by seizures, abnormal movements, focal neurologic deficits, and psychiatric symptoms. A prospective study shows 27% developed AE post herpes simplex virus encephalitis (HSE) in 51 patients; the study also indicated that AE was associated with immune responses against NMDAR and the presence of other antibodies in the neurons around 3 months post HSE (6). Particularly, autoantibodies targeting GABA_B_R, GABA_A_R, GAD65R, anti-alpha-amino-3-hydroxy-5-methyl-4-isoxazolepropionic acid receptor (AMPAR), and unidentified antigens are also reported in previous cases (6, 7). In clinical practice, the atypical early symptoms and false negative results in diagnosis test may lead to the challenge of the diagnosis of AE postinfection (8). The phenomenon of coexistence of multiple anti-neuronal cell surface antibodies following HSE was rarely reported. Our case firstly reports a patient diagnosed with anti-AMPAR encephalitis overlapped with anti-IgLON5 disease post HSE.

## Case description

**Figure 1:**
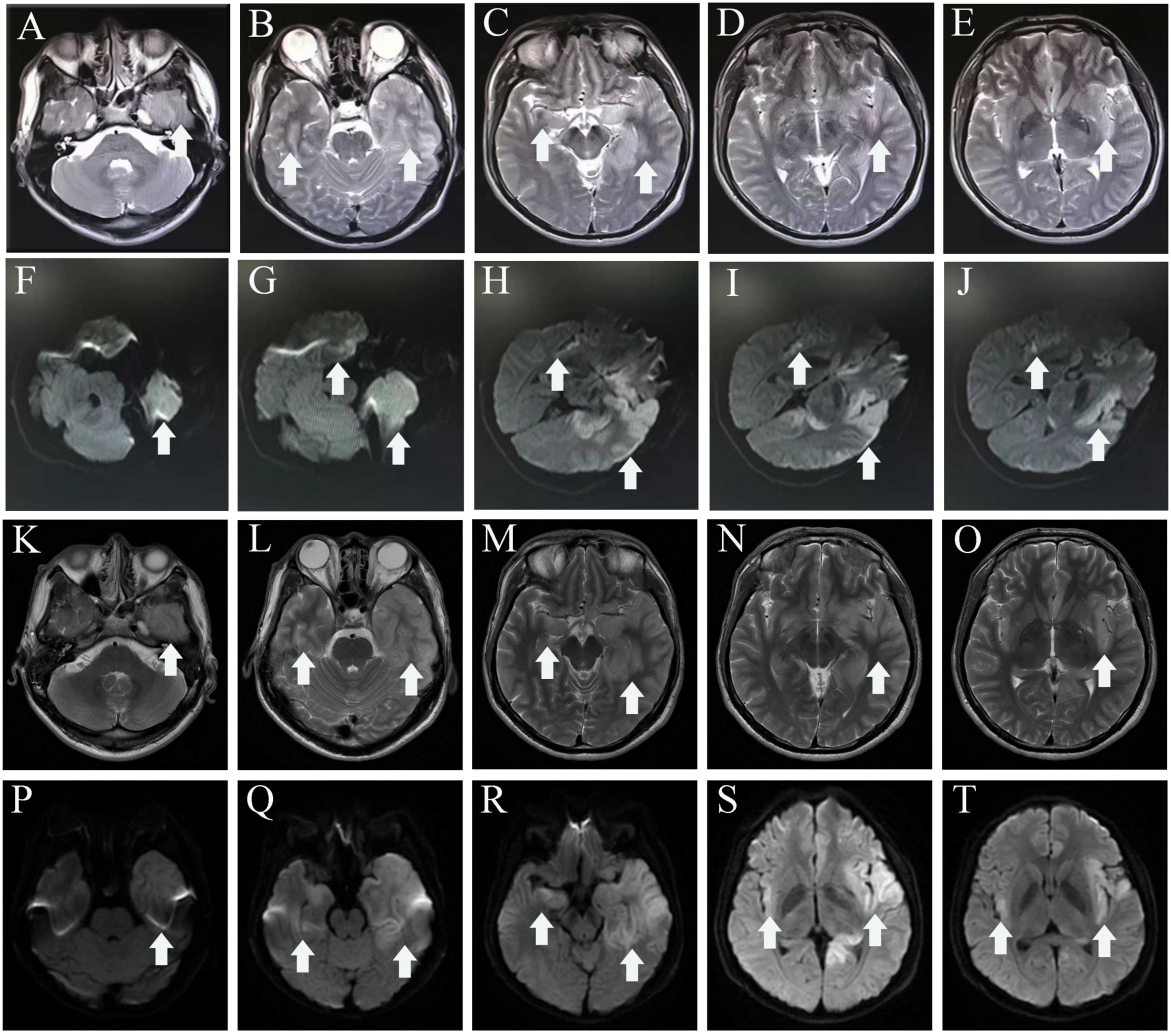
MRI before admission **(A–J)**, axial T2-weighted image **(A–E)**, and diffusion-weighted imaging **(F–J)** show a hyper intense signal in the left frontal lobe, temporal lobe, bilateral insula lobe, and hippocampus. MRI of after IVIG use **(K–T)**, axial T2-weighted image **(K–O)**, and diffusion-weighted imaging **(P–T)** show that the lesions scattered in the left frontal lobe, bilateral temporal lobe, bilateral insula lobe, and hippocampus were not significantly different from before. The white arrowhead showing lesions in MRI. MRI, magnetic resonance imaging.

In mid-July 2022, a previously healthy 30-year-old man experienced acute headache, fever, somnolence for 4 days, and blurred consciousness for 1 day. Four days before admission to the local hospital, the patient had no evident cause of headache and fever. Meanwhile, the body temperature sustained a range of 39.0°C–39.5°C. He also had daily periods of irrepressible need to sleep, and his family-reported total 24-h sleep time was 16 h–18 h. After waking up, he could perform routine activities, such as washing and eating, and then continue to sleep quickly. The aforementioned symptoms were not relieved after using cold remedies (details are unknown). 2 days before admission, nausea with non-ejection vomiting, poor reaction, and blurred consciousness appeared. His sleepiness was worsening, and he could barely recognize his families. Serological tests for blood routine examination showed elevated leukocyte count: 17.15 × 10^9^/L [normal range (NR) (4–10), × 10^9^/L] and neutrophil count: 15.56 × 10^9^/L [NR, (1.6–7.3) × 10^9^/L]. Creatine kinase and creatine kinase isozyme was tested above 40,000 U/L (NR, 25.0 U/L–200.0 U/L) and 6,000 U/L (NR, <25.0 U/L), respectively. Analysis of cerebrospinal fluid (CSF) showed a slightly elevated protein level of 480 mg/L (NR, 80 mg/L–430 mg/L) and mild pleocytosis [white blood cell (WBC) count of 34 × 10^6^/L [NR, (0–8) × 10^6^/L]. The glucose level was normal, with high initial cranial pressure (290 mmH_2_O) and high final cranial pressure (200 mmH_2_O). Brain magnetic resonance imaging (MRI) revealed multiple lesions scattered in the left frontal lobe, bilateral temporal lobe, bilateral insula lobe, and hippocampus showing hyperintensity on T2-weighted and diffusion weighted imaging (DWI) (**Figures 1A–J**). Given anti-infection (cefoperazone sulbactam sodium), the body temperature still fluctuated above 39.0°C. Moreover, his families complained that the patient could not recognize them, had difficulty eating by himself, and experienced urinary incontinence, so he was referred to our hospital.

On admission, the body temperature remained at 39.1°C. The left oral herpes represented 3.0 cm × 2.0 cm with a small amount of white secretion and bleeding when touching. The patient had no history of recurrent oral ulcers. A little of wet rales can be heard in both lungs. An indwelling catheter was in place, accompanied by dark-brown urine. On neurological examination, the level of consciousness stated drowsiness. Stiff neck was found (three fingers under the chin apart from the chest) with positive Kernig’s and Brudzinski’s sign. In the meantime, a modified Rankin scale (mRS) score was 5 (NR, 0); the patient was unable to complete the Mini-Mental State Examination (MMSE).

Urine routine imparted 3+ proteinuria, and the urine was stained dark brown. Creatine kinase showed a higher level of 55,710 u/L (NR, 26 u/L–196 u/L) than before, but a lower level of 182 u/L (NR, <25 u/L) of creatine kinase isozyme. No abnormality was found in blood routine examination, as well as procalcitonin, C-reactive protein, and erythrocyte sedimentation rates; liver and kidney function; tumor series; eight items of infection; blood glucose, tumor marker, folic acid, vitamin B12, anti-nuclear antibody (ANA) spectra; and vasculitis series. Chest computed tomographic scan (CT) did not detect lung original tumors and thymoma. Repeated CSF studies showed a notable elevated WBC count of 88 ×10^6^/L [NR, (0–8) ×10^6^/L] with 80% lymphocytes, an elevated protein level of 655.2 mg/L (NR, 80 mg/L–430 mg/L), and a normal glucose level. Metagenomic next-generation sequencing identified the presence of herpes simplex virus type I (HSV-1) in the CSF with a sequence number of 25900. Conventional electroencephalography (EEG) showed theta activity involving bilateral occipital lobes.

The individual received a 10-day course of intravenous acyclovir sodium (10 mg/kg body weight administered three times daily for 10 consecutive days), in addition to high-dose intravenous methylprednisolone pulse therapy (500 mg/day for 2 days, followed by 1,000 mg/day for 3 days). His body temperature gradually appeared normal, but the level of consciousness remained drowsiness and the communication with others was even difficult because of impaired comprehension. The mRS score was still 5 (NR, 0). Then, a third CSF analysis was performed with normal cranial pressure, and the WBC count was 80 × 10^6^/L [NR, (0–8) ×10^6^/L] with 82% lymphocytes. Additionally, there was a notably increased protein level of 774.5 mg/L (NR, 80 mg/L–430 mg/L), whereas the glucose level remained within the normal range. Results of polymerase chain reaction (PCR) analysis were negative for HSV-1. AE auto-Abs such as anti-NMDAR, LGI1, and AMPAR were screened by cell-based assay (CBA) in the patient’s CSF and/or serum. The full-length human cDNA of each antigen was fused to GFP in the vector pcDNA3.1-C-eGFP by immunofluorescence in HEK293T cells by transfection of the corresponding plasmids. Fluorescence staining was performed by adding 488-labeled goat anti-human IgG secondary antibody (ab97003, Abcam, Cambridge, MA), and immunostaining images were obtained by photographing under an IX73 inverted microscope (Olympus, Tokyo, Japan). Anti-AMPAR-IgG antibodies were detected with a titer of 1:32 in serum but not in CSF. However, there were no antibodies detected against anti-NMDAR, GAD65, IgLON5, GABA_B_R, LGI1, CASPR2, DPPX, GlyR1, mGluR5, DRD2, GABA_A_R, and Neurexin-3αR in the serum and CSF samples. We excluded the following contraindications before IVIG treatment including IgA deficiency, thrombosis, renal dysfunction, and acute renal failure. The patient was administered intravenous immunoglobulin (IVIg) at a dosage of 0.4 g/kg body weight for five consecutive days, along with oral prednisone starting at a daily dose of 50 mg and gradually reducing by 5 mg per week until it was stopped. His condition had been stable until September 2022. Some remarkable improvement was achieved including normal consciousness, regaining functional independence (washing, eating, and dressing). The MMSE score was 6 (reference range, 27–30), and the mRS score (NR, 0) was improved to 3. Repeated brain MRI showed no significant changes in lesions scattered in the left frontal lobe, bilateral temporal lobe, bilateral insula lobe, and hippocampus (**Figures 1K–T**).

**Figure 2:**
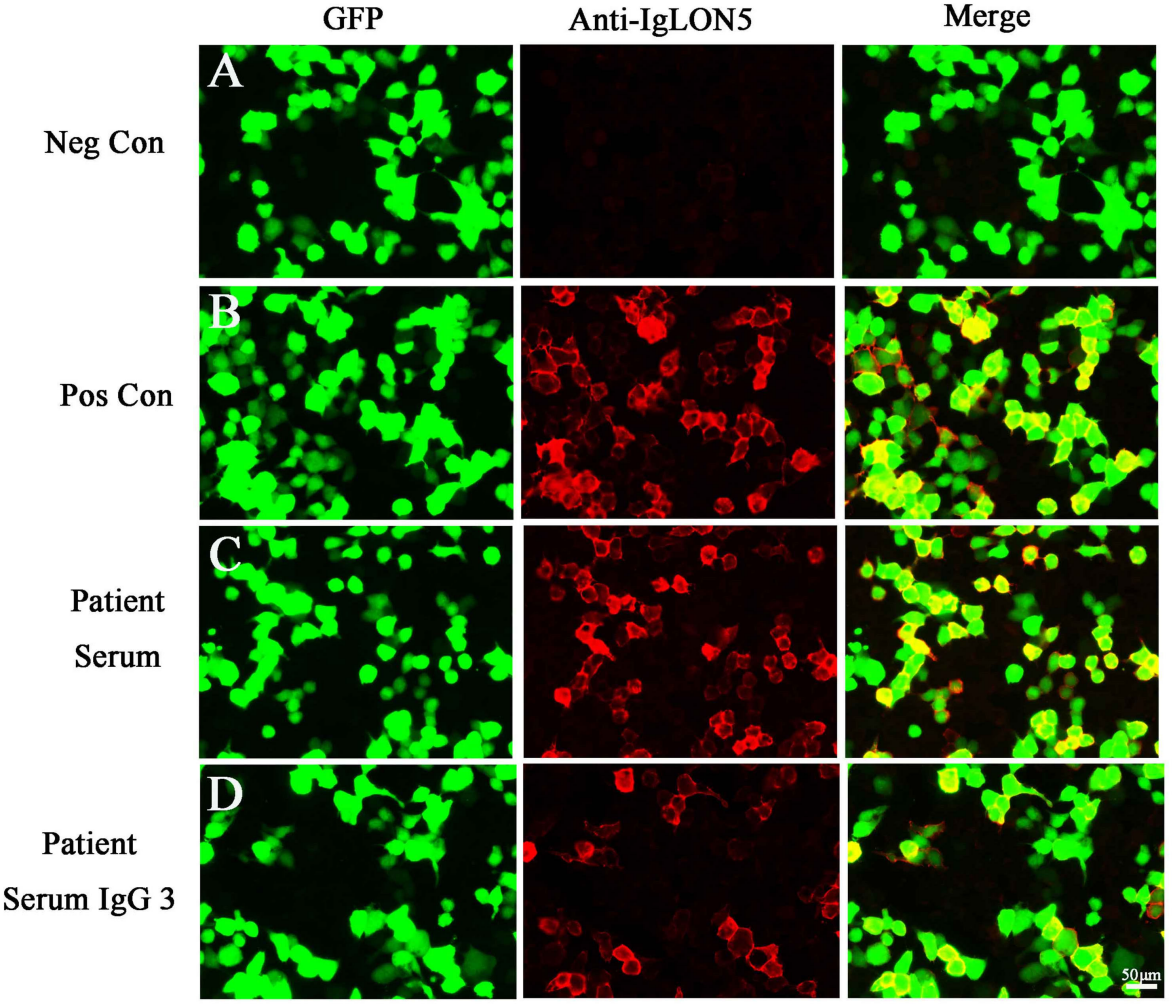
**(A)** Negative controls without IgLON5 antibodies in the serum. **(B)** Positive controls from a patient with classical anti-IgLON5 encephalitis. **(C)** IgLON5-IgG test results (1:30) of the current patient. **(D)** The IgG 3 subtype with IgLON5 test results of the current patient. GFP, green fluorescent protein; Neg Con, negative control; Pos Con, positive control.

**Figure 3:**
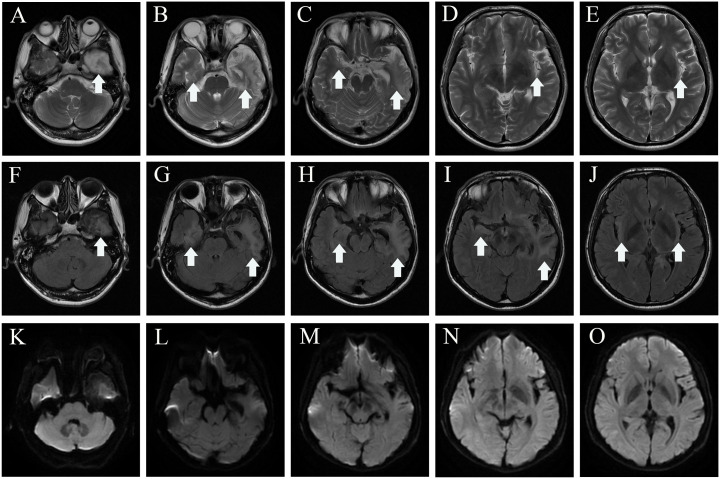
MRI of 2 months after discharge. Axial T2-weighted image **(A–E)**, axial fluid attenuated inversion recovery sequence **(F–J)**, and diffusion-weighted imaging **(K–O)** show the lesions in the left lobe, bilateral temporal lobe, bilateral insula lobe, and hippocampus were reduced, and no new-onset abnormal signal was found on DWI. The white arrowhead showing lesions in MRI. MRI, magnetic resonance imaging.

**Figure 4:**
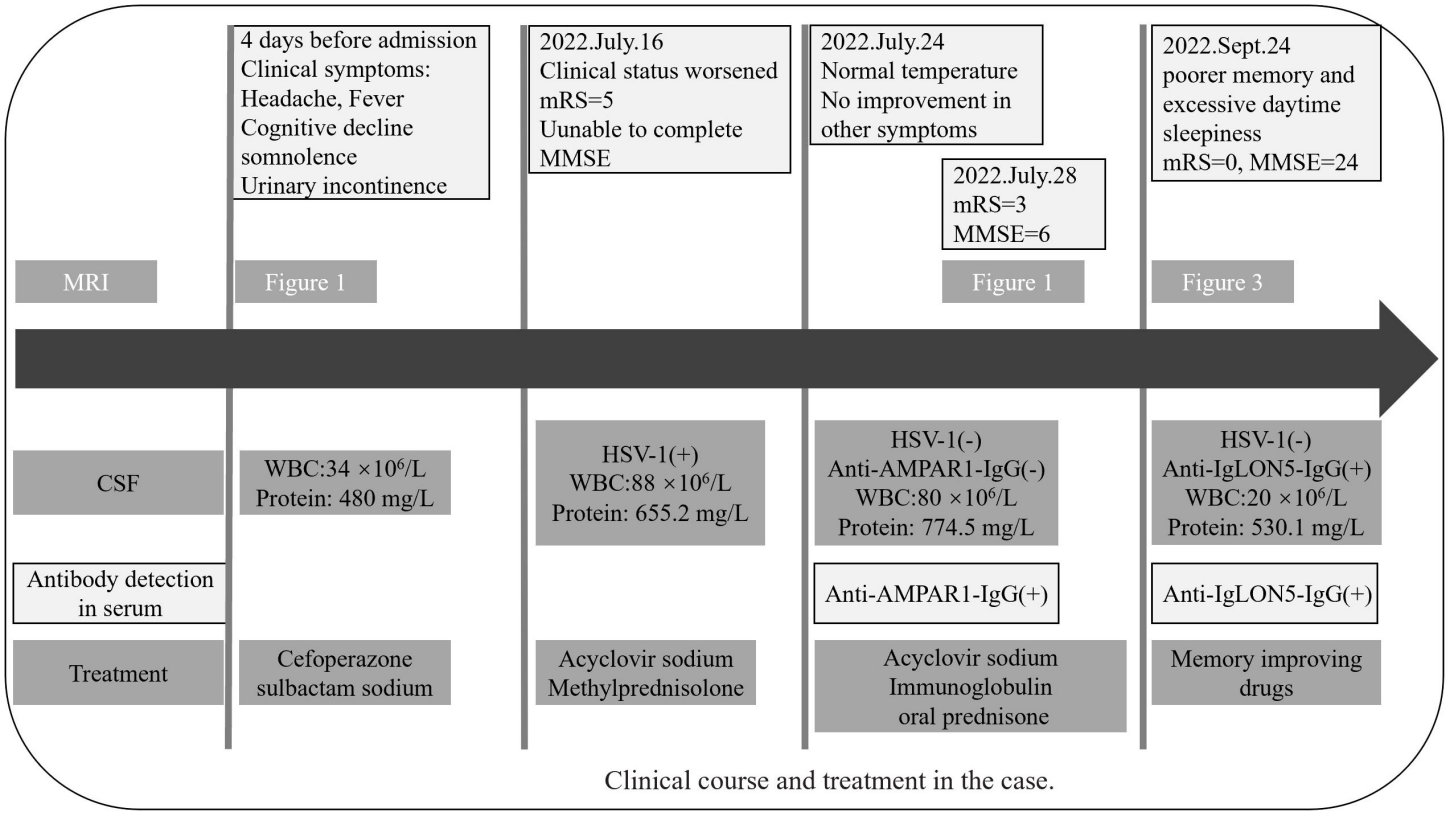
Clinical course and treatment in the case.

Two months later, the patient returned to our medical facility, expressing concerns about his poorer memory and excessive daytime sleepiness (the total sleep time was above 14 h). The MMSE score was 24 (reference range, 27–30), and the mRS score reached the normal level of 0. Repeatability of CSF showed an elevated level of WBC count 20 × 10^6^/L [NR, (0–8) × 10^6^/L] with 93% lymphocytes and a significantly decreased protein level of 530.1 mg/L (NR, 80 mg/L–430 mg/L), which are not normal. Results of PCR analysis were negative for HSV-1. CBA revealed that the anti-IgLON5 antibody tested positive (1:30) in serum and (1:1) in the CSF for antibodies against cell surface proteins, whereas all other antibodies were both negative (**Figure 2**). Additionally, the IgG 3 subtype is predominant in our patients with IgLON5 antibodies that are positive, accompanied by special genetic genes; the human leukocyte antigen (HLA)-DQB1*0301 and HLA-DRB1*0901 results were positive. EEG did not find abnormal activity or an epileptic wave. Repeated MRI showed that the lesions in the left frontal lobe, bilateral temporal lobe, bilateral insula lobe, and hippocampus were reduced and no new-onset abnormal signal was found on DWI (**Figure 3**). In the next half-year of telephone follow-up, the patient’s memory improved gradually with no other complaints, and the patient resumed normal activities of daily living. However, the patient was reluctant to undergo another test for CSF and serum-related autoimmune antibodies. **Figure 4** displays the chronological sequence of the patient’s clinical symptoms and interventions, along with relevant data.

## Discussion

We describe a patient diagnosed with anti-AMPAR encephalitis overlapped with anti-IgLON5 disease after HSV-1 infection. In this case, anti-AMPAR antibodies showed positivity within 2 weeks, whereas anti-IgLON5 antibodies exhibited positivity within 2 months after HSE onset. The appearance of atypical symptoms of HSE underlie an autoimmune etiology, and it led to consideration of virus infection as one of the definite causes of AE (9, 10). Li et al. found parainfectious autoimmunity coexistence of HSV-1, Epstein–Barr virus, human herpes virus 6, cytomegalovirus, varicella zoster virus, and Coronavirus disease 2019 found in an encephalitic patient’s CSF results (1–4), especially that of HSV-1 encephalitis (11). In a prospective observational study, 27% patients with HSE develop anti-NMDAR encephalitis and the clinical symptoms vary with age. Also, similar data confirm that young children have worse outcome and the most manifestation is choreoathetosis (5). Interestingly, our case showed that anti-AMPAR was detected following HSE with cognitive impairment. To our knowledge, there is only one similar case: a 62-year-old female patient developed severe encephalitis and mild improvement with anti-AMPAR encephalitis after 9 weeks of HSV-1 infection (9). Anti-AMPAR encephalitis commonly occurs in the limbic system, especially in the medial temporal lobe, and may also involve the cerebral cortex and cerebellar hemispheres. The typical clinical features are limbic system symptoms, such as cognitive impairment, altered mentation, abnormal behavior, and seizures. Additionally, around 50% of patients with positive AMPAR antibodies had tumors, mainly thymic tumors, lung cancer, and breast cancer (12). In the meantime, our patient was positive for comorbid IgLON5 antibodies in serum and CSF without additional clinical manifestations and intracranial lesions, which may only belong to the “bystander phenomenon” without pathogenic (13, 14). A previous study reported that Epstein–Barr virus infection was a potential trigger, but the mechanisms were not established (2). Also, a strong association with haplotype HLA and IgG subtypes has suggested an underlying autoimmune etiology (15). The IgG 4 subtype is predominant in patients with IgLON5 antibodies that are positive, often accompanied by the special genetic susceptibility genes HLA-DQB1 * 0501 and/or HLA-DRB1 * 1001. However, our patient had the HLA-DQB1 * 0301 and HLA-DRB1 * 0901 alleles and IgG 3 subtype predominance, which were not reported previously. It was speculated that the subtypes and susceptibility genes produced by non-pathogenic anti-IgLON5 antibodies are different from distinct anti-IgLON5 disease.

The synthesis of autoimmune antibodies induced by HSV infection has been clarified through the investigation of various mechanisms. First, molecular simulation mechanisms may be involved such as similarity between NMDAR and HSV proteins (16). Second, neuronal disintegration releases autoantigens after viral infection that disrupt central immune tolerance (17). Furthermore, several investigations have demonstrated that Toll-like receptors (TLRs) 2, 3, and 9 are key factors for the immune recognition of HSV. These receptors of the innate immune system represent as the host’s initial barrier defense mechanism, limiting the spread of viruses and triggering the subsequent activation of the adaptive response. Toll-like receptors or other gene mutations related to the toll-like receptor pathway may be risk factors for HSE and secondary AE post-HSE (18). Except for aforementioned immune mechanisms, several cytokines/chemokines such as interleukin (2, 6, 10), interferon, CXC motif ligand 10, and macrophage inflammatory reaction protein were released after viral infection, These inflammatory cytokines can penetrate the blood–brain barrier and trigger immune response of the central nervous system (19).

Although standardized consensuses or practice guidelines for multiple coexisting antibodies in AE post HSE are lacking, the limited data obtained from retrospective and observational studies suggested that intravenous acyclovir therapy, high-dose corticosteroids (methylprednisolone 1 g intravenously for 3 to 5 days), intravenous immunoglobulin (at a dose of 0.4 g/kg/day for 5 days), plasma exchange, or a combination of treatments should be initiated in a timely manner as the first-line immunotherapies. Additionally, prompt initiation of alternative immunosuppressive medications such as azathioprine, mycophenolate, and rituximab are critical for patients who do not respond well to first-line treatment. In spite of timely prompt identification and early implementation of aggressive immunotherapy, the optimal duration of the first-line immunotherapies remains inconclusive, and more further studies should be confirmed.

In the present case, the patient was admitted to our medical facility because of high fever and neurological symptoms. HSE was initially diagnosed and given antiviral and hormone therapy. The patient’s symptoms continued to deteriorate during hospitalization, except that his temperature dropped to normal. The antibodies related to AE in CSF were reexamined, and the patient was diagnosed with anti-AMPAR encephalitis. After the comprehensive treatment, the patient had a significant recovery. It is worth noting that our case for the first time showed anti-AMPAR overlapping with IgLON5 after HSE, highlighting that the importance in autoantibody detection should be clinically directive for the parainfectious autoimmunity after viral CNS infection (usually HSV-1 encephalitis). However, there is also the limitation that we failed to judge the possibility of AE in time when HSE was diagnosed in the patient, as the likelihood and timing of the emergence of AE post-HSE are uncertain. It is expected that more studies will be conducted to explore the incidence and risk factors of AE post-HSE in the future.

## Conclusion

In conclusion, this is the first reported case of anti-AMPAR1 overlapping with anti-IgLON5 antibodies post HSE. Based on our finding, the parainfectious autoimmunity related to herpes virus and its relevant antibodies have extended far beyond the previously reported adverse outcome of HSE or NMDA receptor encephalitis. Therefore, it is important to clinically detect the presence of autoantibodies as the patient shows neurological relapse or progression after viral infection of the central nervous system (especially HSV-1 encephalitis).

## Patient perspective

The patient and his family were satisfied with the improvement of his clinical condition.

## Data availability statement

The original contributions presented in the study are included in the article/supplementary material. Further inquiries can be directed to the corresponding author.

## Ethics statement

The studies involving humans were approved by Tandu Hospital Ethics Committee. The studies were conducted in accordance with the local legislation and institutional requirements. The participants provided their written informed consent to participate in this study. Written informed consent was obtained from the individual(s) for the publication of any potentially identifiable images or data included in this article.

## Author contributions

SS: Writing – original draft, Writing – review & editing. ZZ: Writing – original draft, Writing – review & editing. JR: Data curation, Formal analysis, Software, Writing – review & editing. XM: Formal analysis, Resources, Writing – review & editing. DS: Data curation, Writing – review & editing. CS: Project administration, Resources, Supervision, Writing – review & editing. XZ: Conceptualization, Data curation, Funding acquisition, Investigation, Project administration, Resources, Supervision, Writing – original draft, Writing – review & editing.
